# Shockwave or Ultrasound Therapy for Tendinopathy? A Systematic Review and Meta-Analysis

**DOI:** 10.3390/jcm15052007

**Published:** 2026-03-05

**Authors:** Artur Dudoń, Magdalena Stania

**Affiliations:** 1 Student Research Club “Impuls”, Department of Theoretical and Practical Basics of Physiotherapy, Faculty of Physiotherapy, Academy of Physical Education, 40-065 Katowice, Poland; 2 Institute of Sport Sciences, Academy of Physical Education, 40-065 Katowice, Poland

**Keywords:** tendinopathy, extracorporeal shock wave therapy, ultrasonics

## Abstract

**Background/Objectives:** This systematic review and meta-analysis was designed to examine the efficacy of extracorporeal shock wave therapy (ESWT) and ultrasound therapy in the treatment of upper and lower limb tendinopathies. **Methods:** The protocol was registered in PROSPERO (CRD420251113976) and conducted in accordance with PRISMA (Preferred Reporting Items for Systematic Reviews and Meta-Analyses) guidelines. Electronic searches were performed in the PubMed, Embase, EBSCOhost, and Ovid MEDLINE databases up to August 2025, to identify randomized controlled trials (RCTs). Mean differences (MDs) and standardized mean differences (SMDs) were calculated with 95% confidence intervals (CIs). Heterogeneity was assessed using the I^2^ statistic, and a random-effects model was applied. Risk of bias was evaluated using the Risk of Bias (RoB 2) tool, and the certainty of evidence was assessed using the Grading of Recommendations Assessment, Development, and Evaluation (GRADE) approach. **Results:** Fourteen RCTs involving 639 patients with tendinopathies were included. All studies were characterized by a high risk of bias. Very low-certainty evidence suggested that ESWT as monotherapy may reduce pain at rest compared with ultrasound therapy in patients with lateral epicondylitis (MD = −1.51; 95% CI: −2.71 to −0.31; *p* = 0.01), although the effect was highly heterogeneous (I^2^ = 89.8%; *p* = 0.002). In patients with upper- and lower-limb tendinopathy, ESWT combined with pharmacotherapy resulted in significantly lower pain intensity compared with ultrasound therapy combined with pharmacotherapy (SMD = −0.6; 95% CI: −1.07 to −0.14; *p* = 0.01). No significant differences in PRTEE (Patient-Rated Tennis Elbow Evaluation) scores were observed between ESWT and ultrasound monotherapy in patients with lateral epicondylitis (MD = −1.06; 95% CI: −11.06 to 8.94; *p* = 0.83; I^2^ = 75.82%), or between ESWT combined with other conservative treatments and ultrasound combined with other conservative treatments (MD = 0.46; 95% CI: −10.22 to 11.15; *p* = 0.93; I^2^ = 0%). **Conclusions:** Very low-certainty evidence suggests that ESWT may be more effective than ultrasound therapy in reducing pain when used as monotherapy in lateral epicondylitis, despite substantial heterogeneity, and when combined with pharmacotherapy in upper- and lower-limb tendinopathies. In terms of function, ESWT appears to provide improvements comparable to those of ultrasound therapy, as assessed by PRTEE scores, in patients with lateral epicondylitis, both as monotherapy and when combined with other conservative treatments. However, significant methodological limitations substantially limit confidence in these findings.

## 1. Introduction

Tendinopathy represents a significant therapeutic challenge in clinical practice. The term “tendinopathy” is commonly used to describe chronic tendon disorders characterized by collagen fiber degeneration and disorganization, and, according to the classical degenerative model, a relative paucity of inflammatory cells and an ineffective healing response [1]. In most cases, tendinopathy is associated with chronic overload, which initiates a cascade of complex and overlapping pathological processes. It manifests as pain, swelling (both localized and diffuse), disruption of tissue architecture, and deterioration of biomechanical function [2].

It is estimated that tendinopathies account for up to 30% of all musculoskeletal consultations in primary care settings [3], and up to 50% of tendon-related injuries in athletic populations [4]. The most prevalent forms of tendinopathy include: rotator cuff tendinopathy, affecting 2.7–10.4% of the general population [5]; patellar tendinopathy, with a reported prevalence of 14% [6]; Achilles tendinopathy, occurring in approximately 23% of individuals [7]; and lateral epicondylopathy, observed in 3–10% of cases [8]. It has been demonstrated that individuals suffering from diabetes, hypercholesterolemia, rheumatic diseases, and renal disorders exhibit a higher prevalence of tendinopathies compared to healthy populations [2]. Other potential risk factors for tendinopathy may include participation in sports (e.g., volleyball and basketball) and occupational activities (e.g., manual labor), both of which involve repetitive movements of the upper and lower limbs that may predispose to tendon injury [9]. The consequences of such overload-induced injuries can significantly disrupt the progression of an athletic career and professional activity, while also limiting the ability to perform daily tasks. Given that tendinopathy is closely associated with repetitive mechanical loading and altered tendon mechanobiology, therapeutic approaches based on mechanical stimulation have been proposed to promote tissue remodeling and pain reduction. However, despite the widespread clinical use of mechanotherapy modalities such as ESWT and ultrasound therapy (UST) [10], uncertainty remains regarding their relative effectiveness and underlying mechanisms of action.

Conservative management of tendinopathy commonly involves mechanotherapy, such as extracorporeal shock wave therapy (ESWT) and ultrasound treatment. ESWT is a non-invasive procedure in which low-frequency mechanical (acoustic) waves act on specific areas of the body to relieve pain and facilitate the healing process [11]. Clinically, ESWT is delivered primarily as either radial or focused shock waves, which differ in pressure amplitude, pulse duration, depth of tissue penetration, and pressure field distribution [12]. Ultrasound therapy (UST) utilizes sound waves beyond the range of human hearing directed into the affected tissues, potentially resulting in thermal effects within the tissue [13]. These effects depend, among other factors, on the mode of ultrasound delivery, including continuous and pulsed emission. Ultrasound therapy is commonly used in physiotherapy practice and is often described as being available in public physiotherapy settings, although access may vary across healthcare systems. Shock wave therapy is frequently reported to be less routinely implemented in some public contexts and more commonly offered in private or specialized clinics; however, availability differs between countries and care models. To date, research evaluating the efficacy of both therapeutic approaches in the treatment of tendinopathy [14,15] has not provided conclusive evidence supporting the superiority of one method over the other.

To the best of our knowledge, no systematic review and meta-analysis has yet provided a head-to-head quantitative comparison of ESWT, including both radial (RSWT) and focused (FSWT) shock wave therapy, versus ultrasound therapy in patients with upper and lower limb tendinopathies, while simultaneously accounting for treatment context and certainty of evidence. Previous reviews have addressed related but distinct questions. Yan et al. [16] and Alharbi [17] evaluated the effects of ultrasonic therapy and ESWT exclusively in patients with lateral epicondylitis; however, their analyses were limited to a single anatomical site and did not include an assessment of the certainty or strength of the evidence. More recently, the systematic reviews and meta-analyses by Majidi et al. [18] and Charles et al. [19] investigated the efficacy of ESWT in patients with various tendinopathies, but ESWT was compared with alternative treatments without ESWT, placebo, eccentric exercise, or other interventions. Importantly, neither review conducted dedicated subgroup analyses directly comparing ESWT with ultrasound therapy.

The novelty of the present review lies in (i) a direct, head-to-head comparison of ESWT and UST across multiple tendinopathy sites; (ii) stratification of included studies according to whether these modalities were applied as monotherapies, as part of combined conservative treatment approaches, or in combination with analgesic and anti-inflammatory medications; (iii) an explicit evaluation of the certainty of evidence in accordance with Evidence-Based Medicine principles. This approach enables a more nuanced interpretation of the available data and supports clinically meaningful recommendations for physiotherapists and orthopedic specialists regarding the use of ESWT and UST in tendinopathy management.

Therefore, the research question was defined using the PICOS format: “In adult patients with upper or lower limb tendinopathy (Population), how effective is shock wave therapy (Intervention) compared to ultrasound therapy (Comparison) in improving primary outcomes—pain intensity (measured using a quantifiable scale, e.g., Visual Analog Scale [VAS] or Numeric Rating Scale [NRS]) and patient-reported physical function and disability—and secondary outcomes such as pressure-pain threshold, muscle strength, range of motion, postural control, adverse effects, and subjective rating of improvement (Outcomes), based on evidence from randomized controlled trials (Study Selection)?”

## 2. Materials and Methods

This systematic review and meta-analysis followed the Preferred Reporting Items for Systematic reviews and Meta-Analyses (PRISMA) 2020 guidelines [20] (Supplementary Table S4). The review protocol was registered on 1 August 2025 in the PROSPERO database with registration number CRD420251113976.

### 2.1. Eligibility Criteria

The articles were reviewed for relevance and selected according to predefined inclusion and exclusion criteria, structured in accordance with the PICOS format.

### 2.2. Population

We included studies with (1) adult patients (aged 18 years or older, or as defined by the study authors); (2) individuals with lower limb tendinopathies, including both insertional and non-insertional Achilles tendinopathy, patellar tendinopathy, proximal hamstring tendinopathy, gluteal tendinopathy, and pes anserine tendinopathy; (3) individuals with upper limb tendinopathies, such as tennis elbow, golfer’s elbow, rotator cuff tendinopathy, extensor carpi ulnaris tendinopathy, De Quervain’s tenosynovitis, and trigger finger (tenosynovitis); (4) cases involving both chronic and acute tendinopathy; (5) tendinopathy of both calcific and non-calcific types; (6) tendinopathy had to be diagnosed based on clinical assessment (e.g., localized tendon pain), with or without imaging confirmation (e.g., ultrasound or MRI), or according to clearly defined diagnostic criteria reported by the trial authors; (7) non-active individuals, as well as recreational and elite athletes. We excluded studies which included patients with: (1) other soft tissue conditions resulting from sports injuries or traumatic events, including muscle disorders, post-traumatic joint stiffness, ligament injuries, and tendon ruptures; (2) neurological conditions, such as nerve entrapments and peripheral nerve injuries; (3) patients who have undergone surgery or have a history of surgical procedures affecting the tendon; (4) systemic diseases with tendon involvement when considered secondary/systemic, such as rheumatoid arthritis or diabetes; (5) pediatric populations (children only); (6) animal or in vitro studies (laboratory-based).

### 2.3. Interventions

We included studies with (1) radial or focused shock wave therapy used as a standalone treatment; (2) radial or focused shock wave therapy combined with other conservative treatment modalities, such as laser therapy, deep friction massage, electrotherapy, autologous blood or platelet-rich plasma injections, injection therapy, or eccentric exercise programs; (3) radial or focused shock wave therapy combined with painkillers or anti-inflammatory drugs. We excluded articles with combined use of radial and focused shock wave therapy within a single treatment session.

### 2.4. Comparisons

Patients in the control groups received either (1) ultrasound therapy used as a standalone treatment; (2) ultrasound therapy combined with another conservative treatment intervention, such as laser therapy, deep friction massage, electrotherapy, autologous blood or platelet-rich plasma injections, injection therapy, or eccentric exercise programs; (3) ultrasound therapy combined with painkillers or anti-inflammatory drugs. We did not include studies where control group participants had been subjected to (1) low-intensity pulsed ultrasound (LIPUS) therapy; (2) surgical treatment; (3) pharmacological treatment alone.

### 2.5. Outcomes

The primary outcomes were as follows: (1) pain intensity measured using a quantifiable scale (e.g., NRS or VAS); (2) patient-reported outcomes related to physical function and disability, evaluated using standardized questionnaires (e.g., quick-disability of the arm, shoulder, and hand (QDASH), Victorian Institute of Sport Assessment–Achilles (VISA-A), Victorian Institute of Sport Assessment–Patella (VISA-P), Patient-Rated Tennis Elbow Evaluation (PRTEE), Short Form-36 (SF-36) health survey). The secondary outcomes included: (3) pressure pain threshold measured by algometer; (4) muscle strength measured with a dynamometer; (5) muscle strength assessed isokinetically; (6) tendon thickness assessed in an ultrasound examination; (7) range of motion; (8) assessment of postural control in static and/or dynamic conditions; (9) subjective improvement, defined as the number of patients reporting self-perceived improvement; (10) treatment success rate assessed using a ranking scale (e.g., the Roles and Maudsley score); (11) adverse effects of shock wave therapy (e.g., transient post-treatment skin redness, bruising, temporary numbness, or hypoesthesia). We included studies with outcomes assessed at short-term follow-up (<12 weeks), mid-term follow-up (3–6 months), and long-term follow-up (>6 months) after treatment completion. We excluded trials only assessing the health economics (e.g., costs of interventions, resource implications).

### 2.6. Study Selection

The selection of articles was guided by the following inclusion criteria: (1) randomized controlled trials (RCTs); (2) full-text of a peer-reviewed original research article; (3) articles published in English or Polish; (4) articles were eligible if they reported at least four predefined application parameters for each therapy (ESWT and ultrasound therapy). For shock wave therapy, the predefined parameters included: number of shocks, frequency, energy flux density or pressure, number of sessions, and treatment frequency. For ultrasound therapy, the predefined parameters included: duty cycle, ultrasound frequency, ultrasound power density, duration of application, number of sessions, and treatment frequency. Studies with missing data were not supplemented by contacting authors and were handled according to these predefined criteria. Retrospective studies, case reports, conference abstracts, proceedings, commentary articles, secondary analyses, systematic reviews, meta-analyses, and narrative reviews were excluded.

### 2.7. Information Sources

Relevant studies were identified by searching the following electronic databases: PubMed, Embase, EBSCOhost, and Ovid MEDLINE. Databases were searched from their inception until the last entry, which was 5 August 2025 for Embase and 6 August 2025 for PubMed, EBSCOhost, and Ovid MEDLINE. To minimize the risk of overlooking relevant sources, we implemented complementary strategies to systematically explore grey literature, including the use of customized Google search engines, targeted websites, and consultation with clinical experts [21]. The bibliographies of all identified articles were systematically reviewed to discover further relevant studies. Additionally, on 7 February 2026, trial registries, including ClinicalTrials.gov and the WHO International Clinical Trials Registry Platform (ICTRP), were systematically searched to identify ongoing or unpublished studies.

### 2.8. Search Strategy

An extensive set of title/abstract keywords was used to comprehensively capture relevant studies, including, among others: tendinopathy, enthesopathy, tendonitis, shock wave, shock-wave, shockwave, shockwaves, extracorporeal, ultrasound, ultrasonic, ultrasonics, and sonotherapy. Furthermore, no controlled vocabulary terms (e.g., MeSH/Emtree) were included in the search strategy. The complete PubMed search strategy is outlined below: (((shock wave[tiab] OR shock-wave[tiab] OR shockwave[tiab] OR shockwaves[tiab] OR extracorporeal[tiab])) AND (ultrasound[tiab] OR ultrasonic[tiab] OR ultrasonics[tiab] OR sonotherapy[tiab]) AND (tendinopathy[tiab] OR enthesopathy[tiab] OR tendonitis[tiab] OR tendonopathy[tiab] OR tendinosis[tiab] OR epicondylitis[tiab] OR tennis elbow[tiab] OR golfer’s elbow[tiab] OR lateral epicondylitis[tiab] OR medial epicondylitis[tiab] OR humeral epicondylitis[tiab])). Detailed search strategies for each database, including applied filters and restrictions, are documented in Supplementary Table S1.

### 2.9. Selection and Data Collection Processes

Study selection was conducted through a two-phase screening process. In the initial phase, titles and abstracts were evaluated against predefined eligibility criteria to identify potentially relevant articles and reduce the inclusion of non-pertinent studies. In the subsequent phase, full-text articles of those meeting the initial criteria were thoroughly assessed for final inclusion. Two authors (AD and MS) independently screened studies and extracted data, remaining unaware of each other’s decisions during the initial selection stage; any disagreements were resolved by discussion and consensus.

### 2.10. Data Items and Study Quality

The following data items were sought: basic publication characteristics (first author, publication year, country), participant characteristics (age, type of tendinopathy, duration of symptoms, number of patients), interventions (for shock wave therapy: type of shock wave therapy, number of shocks, frequency, energy flux density or pressure, number of sessions, and frequency of treatment; and for ultrasound therapy: duty cycle, ultrasound frequency, ultrasound power density, duration of application, number of sessions, and treatment frequency), outcomes (primary and secondary outcomes, methods and timings of assessments), and results.

The methodological quality of the RCTs was assessed using the Physiotherapy Evidence Database (PEDro) scale. This instrument, which scores studies on a scale from 1 to 10, is recognized as a reliable and valid tool for evaluating the quality of RCTs [22]. Based on the PEDro score, the methodological quality of trials was rated as high (PEDro scores ≥ 7), medium (PEDro scores of 4 to 6), or low (PEDro scores ≤ 3). The methodological quality of the articles included was assessed independently by two reviewers (AD and MS). In cases of divergent assessments, the final decision was made through joint discussion between the two authors.

The internal validity of the selected studies was evaluated using the Risk of Bias (RoB) 2 tool for RCTs [23]. Assessment focused on five key domains: bias related to the randomization procedure, bias due to deviations from the intended interventions, bias resulting from incomplete outcome data, bias in the measurement of outcomes, and bias associated with selective reporting of results. Each study was classified within these five domains as having a ‘low,’ ‘high,’ or ‘unclear’ risk of bias.

Assessment of the strength of the evidence was conducted using the guidelines established by the Grading of Recommendations Assessment, Development, and Evaluation (GRADE) methodology [24]. This approach uses four categories (very low, low, moderate, and high) to rate the quality of the evidence available for selected outcomes. We used the GRADEpro Guideline Development Tool (McMaster University (Ontario, Canada), 2015; developed by Evidence Prime, Inc. (Kraków, Poland); available from gradepro.org) [25] to generate a summary of findings table.

### 2.11. Synthesis Methods

Data from individual studies were combined in a meta-analysis only when deemed appropriate. For each outcome, we included a single prespecified effect size per trial, selecting the time point closest to the end of treatment. To account for clinical heterogeneity, separate meta-analyses were predefined and conducted according to intervention context: (1) studies in which ESWT or ultrasound therapy was used as monotherapy for tendinopathy; (2) studies in which ESWT or ultrasound therapy was used in combination with other conservative therapies for tendinopathy; (3) studies in which ESWT or ultrasound therapy was used in combination with painkillers and/or anti-inflammatory drugs for tendinopathy. For continuous outcomes, when all included primary studies employed the same measurement scale, the mean difference (MD) with a 95% confidence interval (CI) was reported. When different measurement scales were used to assess the same outcome, the standardized mean difference (SMD) was applied. For pain outcomes, all included studies in which ESWT or ultrasound therapy was used in combination with painkillers and/or anti-inflammatory drugs assessed pain intensity using either the VAS or the NRS, both reported on a common 0–10 scale. Therefore, no scale transformation was required. Heterogeneity was assessed using the I^2^ statistic, with thresholds of 25% indicating low, 50% moderate, and 75% high heterogeneity. A random-effects approach was selected because substantial clinical heterogeneity was anticipated due to variations in study design, interventions, outcome measures, and trial settings. When sufficient data were available, subgroup analyses were performed according to type of ESWT (RSWT vs. FSWT), and ultrasound mode (continuous vs. pulsed). Statistical significance was defined as *p* < 0.05. Statistical analyses were performed using Statistica Software (Data Analysis Software System, serial number Statistica AWF Katowice: JPZ009K288211FAACD-Q, version 13.3, Plus Set package).

## 3. Results

### 3.1. Study Selection

**Figure 1:**
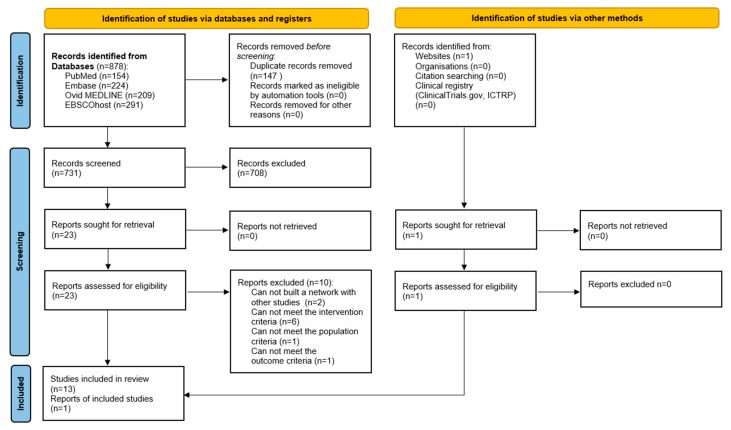
PRISMA (Preferred Reporting Items for Systematic Reviews and Meta-Analyses) 2020 flow diagram.

A total of 879 publications were retrieved through the electronic database search. The full texts revealed that only 14 met the inclusion criteria (Figure 1) and included 639 patients with upper or lower limb tendinopathy. Three articles [14,26,27] reported distinct patient outcomes derived from the same research project, which included a total of 26 participants. In four studies (*n* = 180 patients) the authors compared efficacy of ESWT and UST as monotherapies [15,28,29,30], and in three (*n* = 117 patients) they contrasted the efficacy of combined ESWT and UST with other conservative treatment methods for tendinopathy [31,32,33]. In contrast, seven studies (*n* = 342 patients) included an evaluation of combined ESWT and UST with painkillers and anti-inflammatory drugs [14,26,27,34,35,36,37].

### 3.2. Study Characteristics and Quality Assessment

All studies were performed within a single research center [14,15,26,27,28,29,30,31,32,33,34,35,36,37]. The included RCTs were performed in the following countries: eight in Poland [14,26,27,28,29,33,34,37], three in Turkey [15,30,31], and one each in Italy [35], Greece [36], and India [32].

Reported recruitment periods indicated no overlap between most included trials. Three publications from the same project [14,26,27] shared an identical recruitment period and were considered to derive from a single participant cohort. In two other studies [15,28], recruitment periods partially overlapped; however, these trials were conducted in different research centers and countries. Based on the available data, no additional evidence of participant overlap was identified.

The main aspects of each intervention, along with the major outcomes of the included studies that used ESWT and UST as monotherapy, are presented in Table 1. Studies that used ESWT and UST as combined therapy are shown in Table 2, and those that used ESWT and UST in conjunction with painkillers or anti-inflammatory drugs are summarized in Table 3.

**Figure 2:**
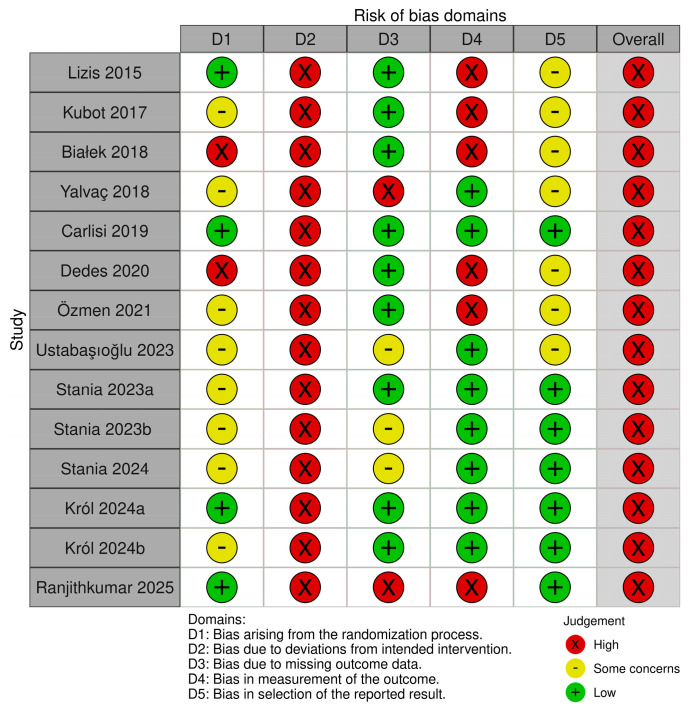
“Traffic light” plots of the domain-level judgements for each individual result [14,15,26,27,28,29,30,31,32,33,34,35,36,37].

**Figure 3:**
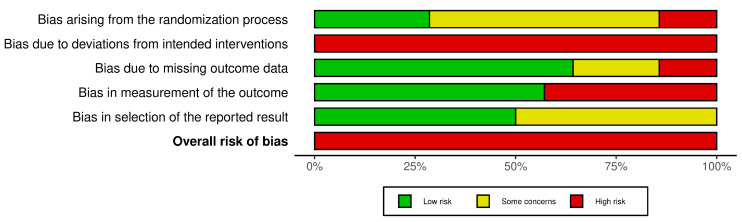
Weighted bar plots of the distribution of risk-of-bias judgements within each bias domain.

Among the fourteen studies included in this systematic review, the randomization process was assessed as low risk of bias in four studies [28,32,35,37], high risk in two studies [29,36], and with some concerns in eight studies [14,15,26,27,30,31,33,34]. Since none of the fourteen studies implemented blinding of patients or therapists, all were rated as having a high risk of bias for deviations from the intended intervention. Missing outcome data were judged to pose a low risk of bias in nine studies [26,28,29,31,33,34,35,36,37] and a high risk in two studies [15,32]. The risk of bias related to outcome measurement was classified as low in eight studies [14,15,26,27,30,33,35,37] and high in six studies [28,29,31,32,34,36]. With respect to the selection of reported results, seven studies were assessed as low risk [14,26,27,32,33,35,37], while seven studies raised some concerns [15,28,29,30,31,34,36]. The overall risk of bias in the included studies is illustrated in Figure 2 and Figure 3.

Supplementary Table S2 provides an overview of the methodological quality of the RCTs assessed using the PEDro scale. The methodological quality of these trials was rated as high [14,26,27,28,32,33,35,37] or medium [15,29,31,34,36]. None of the submissions were evaluated as low. However, despite relatively high PEDro scores, the RoB 2 assessment identified a high risk of bias in several domains across all included studies, particularly due to the lack of blinding of patients and therapists (Figure 2). As none of the trials implemented blinding, all were judged to have a high risk of bias for deviations from the intended interventions, which may especially affect subjective outcomes such as pain and patient-reported measures.

### 3.3. Publication Bias

Funnel plots and Egger’s regression test were planned to assess publication bias when at least 10 studies were available per outcome. This threshold was not met for any of the analyses: rest pain with ESWT and ultrasound as monotherapies (k = 2), pain with ESWT and ultrasound combined with painkillers and anti-inflammatory drugs (k = 3), PRTEE score with ESWT and ultrasound as monotherapies (k = 2), and PRTEE score with ESWT and ultrasound combined with other conservative therapies (k = 2). Therefore, formal statistical assessment of publication bias was not performed.

### 3.4. Results of Individual Studies and Syntheses

**Figure 4:**
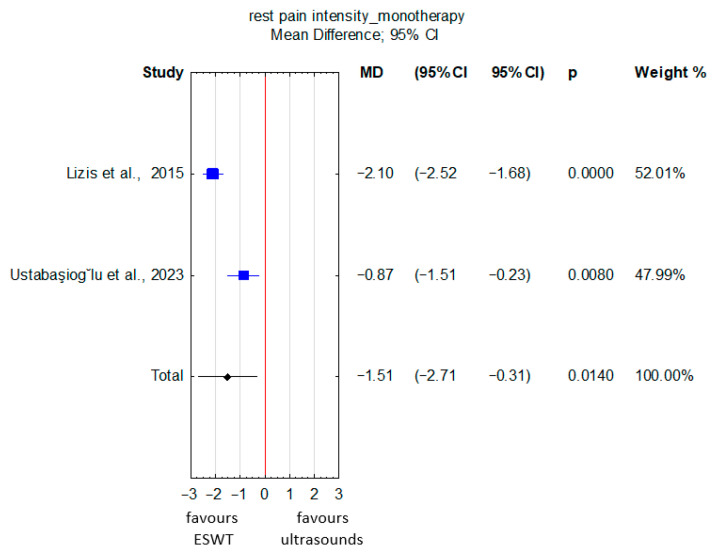
Forest plot comparing rest pain intensity between ESWT and ultrasound therapy used as monotherapies in patients with lateral epicondylitis [28,30]. The red vertical line represents the line of no effect (MD = 0), indicating no difference between the compared groups.

The evidence, rated as very low quality (Supplementary Table S3), suggested that ESWT administered as monotherapy significantly reduced the pooled intensity of rest pain, assessed using the VAS, compared with ultrasound therapy alone in patients with lateral epicondylitis (MD = −1.51; 95% CI: −2.71 to −0.31; *p* = 0.01) (Figure 4). However, heterogeneity across the included studies was high (I^2^ = 89.8%, *p* = 0.002), and the risk of bias in these studies was considered very serious.

**Figure 5:**
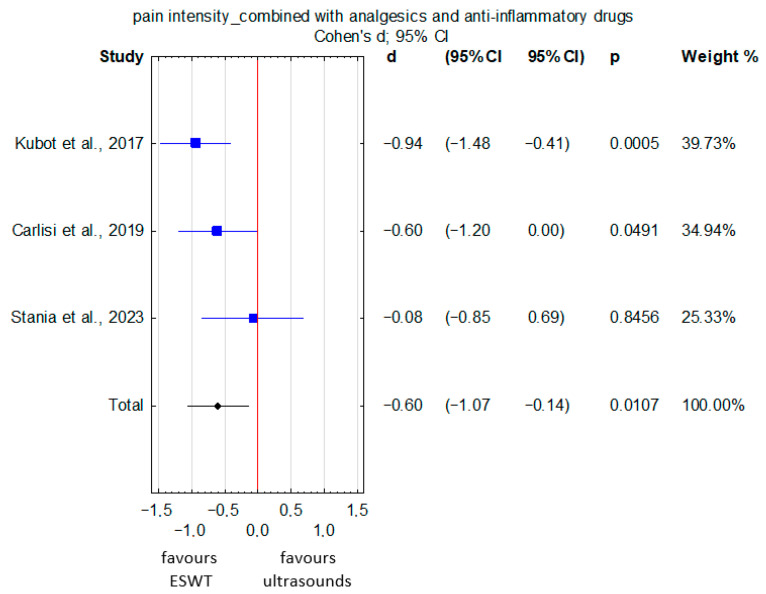
Forest plot comparing pain intensity between ESWT and ultrasound therapy, both combined with analgesics and anti-inflammatory drugs, in patients with upper- and lower-limb tendinopathy [26,34,35]. The red vertical line represents the line of no effect (MD = 0), indicating no difference between the compared groups.

In patients with upper- and lower-limb tendinopathy receiving ESWT in combination with analgesics and anti-inflammatory drugs, the pooled pain intensity, assessed using VAS and NRS, was significantly lower compared with those treated with ultrasound therapy combined with pharmacotherapy (SMD = −0.6; 95% CI: −1.07 to −0.14; *p* = 0.01) (Figure 5). There was no significant heterogeneity among the studies (I^2^ = 39.55%, *p* = 0.19). However, the quality of evidence was rated as very low (Supplementary Table S3) due to very serious risk of bias and serious imprecision. In the subgroup analysis, FSWT combined with analgesics and anti-inflammatory drugs resulted in a statistically significant reduction in pain intensity compared with ultrasound therapy combined with analgesics and anti-inflammatory drugs (SMD = −0.60; 95% CI: −1.20 to 0.00; *p* = 0.049), whereas RSWT combined with analgesics and anti-inflammatory drugs did not differ significantly from ultrasound therapy combined with analgesics and anti-inflammatory drugs (SMD = −0.56; 95% CI: −1.40 to 0.29; *p* = 0.2) (Supplementary Figure S1). Furthermore, subgroup analysis by ultrasound mode demonstrated a statistically significant reduction in pain intensity only for continuous ultrasound combined with analgesics and anti-inflammatory drugs (SMD = −0.60; 95% CI: −1.20 to 0.00; *p* = 0.049) (Supplementary Figure S2).

**Figure 6:**
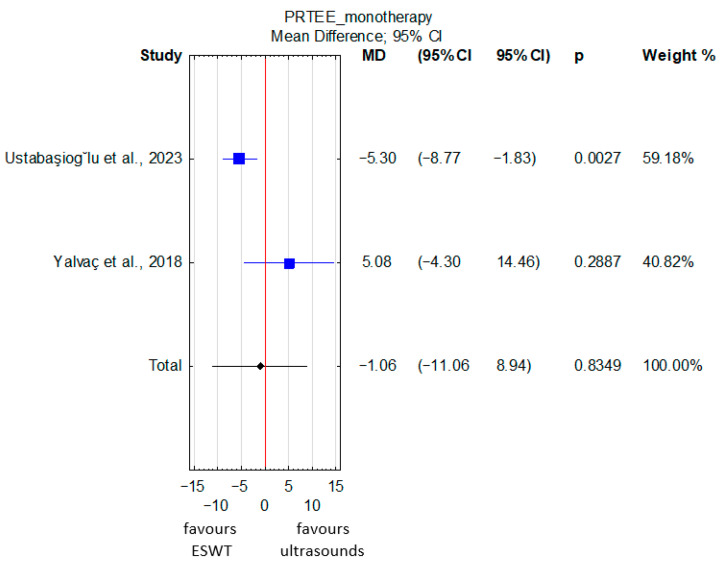
Forest plot of PRTEE scores between ESWT and ultrasound therapy administered as monotherapies in patients with lateral epicondylitis [15,30]. The red vertical line represents the line of no effect (MD = 0), indicating no difference between the compared groups.

A comparison of pooled PRTEE scores in patients with lateral epicondylitis revealed no significant differences between ESWT and ultrasound therapy as monotherapies (MD = −1.06; 95% CI: −11.06 to 8.94; *p* = 0.83) (Figure 6). Considerable heterogeneity was observed (I^2^ = 75.82%, *p* = 0.04). The certainty of evidence was again rated as very low (Supplementary Table S3) due to a very serious risk of bias in the included studies, as well as serious inconsistency and imprecision.

**Figure 7:**
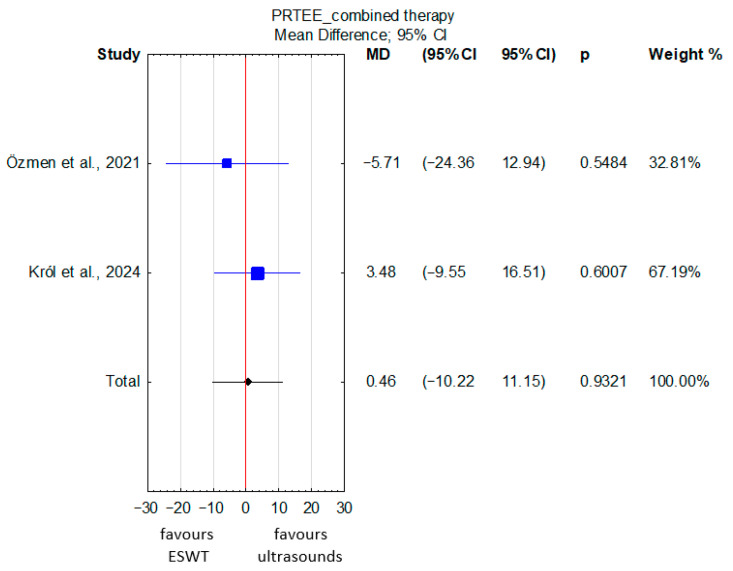
Forest plot of PRTEE scores comparing ESWT and ultrasound groups administered in combination with other conservative therapies in patients with lateral epicondylitis [31,33]. The red vertical line represents the line of no effect (MD = 0), indicating no difference between the compared groups.

Patients with lateral epicondylitis undergoing ESWT combined with other conservative therapies achieved comparable PRTEE scores to those receiving ultrasound therapy combined with conservative treatments (MD = 0.46; 95% CI: −10.22 to 11.15; *p* = 0.93) (Figure 7), although the certainty of the evidence was rated as very low due to a very serious risk of bias and serious imprecision (Supplementary Table S3). There was no significant heterogeneity among the studies.

## 4. Discussion

The available data suggest that pain reduction at rest in patients with lateral epicondylitis is greater following ESWT used as monotherapy than after ultrasound therapy used as monotherapy; however, the effect was highly heterogeneous and should not be overgeneralized. When combined with pharmacotherapy, ESWT may significantly reduce pain intensity in patients with upper- and lower-limb tendinopathy compared with ultrasound therapy. Functional outcomes, as measured by PRTEE, were comparable between ESWT and ultrasound therapy in patients with lateral epicondylitis, both when used as monotherapy and in combination with other conservative treatments. According to the GRADE assessment, all outcomes were supported by very low-certainty evidence, mainly due to very serious risk of bias and serious imprecision, and the results should therefore be interpreted with caution. Overall, these findings suggest that ESWT may provide some clinical benefits for pain reduction in tendinopathy; however, the evidence is limited, and robust, high-quality studies are needed to confirm or refute these observations.

The high heterogeneity observed in the meta-analysis for rest pain intensity and PRTEE scores, as indicated by an elevated I^2^ value, may be attributed to methodological and clinical variability across the included studies [15,28,30]. Specifically, variability in ESWT protocols applied as monotherapy (including the number of shock waves, pulse frequency, and number of sessions), as well as heterogeneity in ultrasound therapy parameters used as monotherapy (such as power density and treatment frequency), may have contributed to the observed inconsistency. Additional clinical differences, including patient age and duration of symptoms, may have further influenced the dispersion of effect estimates.

Available meta-analyses [16,17] suggest that ESWT may provide greater pain reduction than ultrasound therapy in patients with lateral epicondylitis, while evidence for functional outcomes remains less consistent. This apparent emphasis on tennis elbow in prior and comparative analyses is intentional and reflects the fact that lateral epicondylitis constitutes the most extensively studied tendinopathy in the current evidence base comparing the efficacy of ESWT with ultrasound therapy. Alharbi et al. [17] reported significantly greater pain improvement in favor of ESWT, although no significant differences were observed in functional outcomes assessed using the PRTEE, alongside substantial heterogeneity across included studies. Similarly, the meta-analysis by Yan et al. [16] demonstrated the superiority of ESWT in pain reduction at follow-up periods ranging from 1 to 6 months and improvements in grip strength at 3 months; however, no statistically significant between-group differences were found for several functional outcomes. Consequently, while our review encompasses tendinopathies of both the upper and lower limbs, the comparative discussion necessarily centers on lateral epicondylitis due to its dominant representation and evidentiary weight in the existing literature.

In our review, ESWT and UST were applied with varying technical parameters, which may help explain some of the observed heterogeneity in rest pain intensity and PRTEE scores when both shockwave and ultrasound therapies were used as monotherapies. In the treatment of various upper and lower limb tendinopathies, RSWT was used more frequently than FSWT in the included studies [14,26,27,29,34,37], likely affecting larger tissue volumes but delivering lower focal energy, whereas FSWT concentrated energy in smaller regions. The majority of investigators employed an energy flux density of 0.20–0.22 mJ/mm^2^ [31,32,33] and a pressure of 2.5–3 bar [26,29,30,37], most often delivering 2000 shocks [15,30,32], with treatments typically administered in three weekly sessions [15,27,35,37]. Differences in these parameters across studies may contribute to variability in treatment efficacy. For UST, therapy most commonly consisted of 10 sessions [31,33,34,35], with power densities of 0.5 W/cm^2^ [29,33,34] or 1.5 W/cm^2^ [15,30,35], and ultrasound emission either continuous [15,28,30] or pulsed with a 20% duty cycle [29,32,33]. Variations in these intensity and duty cycle parameters may plausibly explain some of the differential effects observed between ESWT and UST in patients with upper and lower limb tendinopathies.

Subgroup analyses indicated that the reduction in pain intensity in patients with upper- and lower-limb tendinopathy was significantly greater for FSWT combined with pharmacotherapy compared with the ultrasound group. Based on the available literature, across different types of tendinopathies, both RSWT and FSWT demonstrate comparable efficacy in improving patients’ quality of life [38]. In rotator cuff tendinopathy, FSWT showed slightly better long-term pain reduction, whereas both modalities exhibited similar efficacy in functional improvement [39]. FSWT, however, demonstrated superiority over RSWT in enhancing function in patients with plantar fasciitis [40]. In lateral epicondylitis, both methods produced very similar outcomes, increasing wrist flexor and extensor strength and reducing pain symptoms [41] ESWT is considered a safe therapeutic method, although it may be accompanied by adverse effects [42]. The most commonly reported side effects include subcutaneous hematoma, swelling, and skin erythema [33,43].

The mechanisms of action of mechanical waves, such as shockwave and ultrasound, on tendon tissue are complex. The energy of a shockwave can penetrate deeper tissues compared to ultrasound [1]. The mechanism of action of ESWT in the treatment of tendinopathy involves the induction of microinjuries within tissues, which stimulate reparative processes, angiogenesis, tissue regeneration, and analgesic effects through pain modulation mediated by nociceptor stimulation [44,45]. The regenerative processes of the tendon following shockwave application are also attributed to the stimulation of tenocyte proliferation and collagen synthesis [46,47]. UST primarily utilizes the thermal effect, which increases the extensibility of fibrous tissue, accelerates tissue metabolism, and elevates the patient’s pain threshold [48]. Ultrasound waves also enhance the expression of type I and type III collagen [49] as well as the migration and proliferation of tendon tissue cells [50], processes that play a crucial role in the physiological healing of tendons [51].

The therapeutic effects of shockwave treatment on tendons occur gradually over time [52,53]. Prolonged processes of new collagen synthesis within the injured tendon [54], its reorganization and remodeling [55], as well as sustained neovascularization [56], may explain the progressive and delayed beneficial outcomes of shockwave therapy observed in patients with tendinopathy. Compared to ESWT, UST requires a greater number of treatment sessions [57].

Both ESWT and UST can be applied as monotherapies [15,28,29,30] or as combined therapies with other treatment modalities [34,35,36]. As these combined approaches involved heterogeneous co-interventions, including other conservative treatment modalities or pharmacotherapy, the observed effects cannot be attributed to any specific adjunct and may reflect co-intervention effects rather than ESWT or UST alone; therefore, they should not be interpreted as supporting a standardized combined treatment protocol.

This systematic review and meta-analysis has several limitations. A major limitation is the limited number of high-quality randomized controlled trials, most of which had relatively small sample sizes. In addition, none of the included studies implemented blinding of participants or therapists and many lacked blinding of outcome assessors for subjective outcomes such as pain intensity and PRTEE scores, thereby increasing the risk of performance and detection bias. Another important limitation is the substantial heterogeneity among studies assessing the efficacy of ESWT and ultrasound therapy as monotherapies, resulting from differences in therapeutic protocols, follow-up durations, and patient characteristics, which complicates the interpretation of pooled estimates. Furthermore, we did not include a controlled vocabulary component (e.g., MeSH/Emtree terms) in our search strategy, which may have limited the comprehensiveness of study identification. All outcomes were supported by very low-certainty evidence according to the GRADE assessment, reflecting cumulative methodological limitations rather than statistical imprecision alone. Finally, the exclusion of studies published in languages other than Polish and English may have introduced language bias.

## 5. Conclusions

Very low-certainty evidence suggests a possible association between extracorporeal shockwave therapy and greater pain reduction compared with ultrasound therapy when used as monotherapy in lateral epicondylitis, although the findings were highly heterogeneous. A similar potential association was observed when ESWT and ultrasound therapy were combined with pharmacological co-interventions in upper- and lower-limb tendinopathies; however, these co-interventions were not standardized across studies. In terms of function, ESWT appears to provide improvements comparable to those of ultrasound therapy when assessed by PRTEE scores in patients with lateral epicondylitis, both when used as monotherapy and when combined with other conservative treatments. Given the substantial methodological limitations and the very low certainty of the evidence, these findings should not be considered clinically directive. Further well-designed randomized controlled trials with standardized interventions and clearly defined follow-up periods are required to clarify the comparative effects of ESWT and ultrasound therapy in tendinopathies.

## Figures and Tables

**Table 1:** Characteristics of included studies in which ESWT and ultrasound therapy were used as monotherapy for tendinopathy.

Reference	Diagnosis	Groups	ESWT Intervention	UST Intervention	Outcome Measure	Follow-Up	Results
Lizis et al., 2015 [28] (Poland)	Lateral epicondylitis Duration (months): >12 Group I: 14.9 ± 2.1 Group II: 15.1 ± 1.9	Group I (*n* = 25): ESWT Age (years): 47.9 ± 4.4 Group II (*n* = 25): UST Age (years): 49.0 ± 4.5	ESWT: 1000, 1500, and 2000 shocks during the first, second, and third through fifth treatments—8 Hz, 2.5 bars, 0.4 mJ/mm^2^, 5 sessions, once a week Site: the area with the most intense pain	1 MHz, 0.8 W/cm^2^, continuous mode, <10 min, 10 sessions, three times a week Site: along the extensor tendons	pain (VAS), pain of the affected upper limb during gripping (Martin Vigorimeter), resting pain, pain felt during palpation of the LE of the humerus, and pain during the Thomsen test	post-treatment and 3 months after the final treatment session	ESWT decreased pain to a significantly greater extent than UST immediately and 3 months post-treatment.
Białek et al., 2018 [29] (Poland)	Lateral epicondylitis Duration (months): >3 Group I: 8.7 ± 9.7 Group II: 8.1 ± 8.9	Group I (*n* = 13): RSWT Age (years): 45.1 ± 7 Group II (*n* = 13): UST Age (years): 45.1 ± 8.8	RSWT: 4000 shocks—8 Hz, 2.5 bars, 3 sessions, once a week. Site: to the most painful point on the lateral humeral epicondyle and along the extensor tendons	1 MHz, 0.5 W/cm^2^, pulsed mode (20%), 10 min, ten sessions held on weekdays over 2 weeks Site: to the most painful point on the lateral humeral epicondyle and along the extensor tendon	rest pain, night pain and pain during activity (VAS), hand grip strength (hand dynamometer), treatment efficacy (Roles-Maudsley scale)	1, 3 and 6 weeks after the final treatment session	RSWT and UST effectively reduce pain and improve grip strength in lateral epicondylitis. The therapeutic effect is comparable in both groups
Yalvaç et al., 2018 [15] (Turkey)	Lateral epicondylitis Duration (months): >3 Group I: 8.2 ± 3.6 Group II: 7.9 ± 3.3	Group I (*n* = 20): ESWT Age (years): 46.04 ± 9.24 Group II (*n* = 24): UST Age (years): 43.75 ± 4.52	ESWT: 2000 shocks—10–15 Hz, 1.5–2.5 bars, 3 sessions, once a week. Site: in painful area	1 MHz, 1.5 W/cm^2^, continuous mode, 5 min, ten sessions held on weekdays over 2 weeks Site: in painful area	PRTEE scores (pain, function), pain (VAS), grip strength evaluation (Baseline Hydraulic Hand Dynamometer), functional status evaluation (DASH/quick- DASH), Quality of life evaluation (SF-36)	post-treatment and 1 month after the final treatment session	ESWT and therapeutic UST are equally effective in treating LE. ESWT is an alternative therapeutic intervention and as effective as UST
Ustabaşıoğlu et al., 2023 [30] (Turkey)	Lateral epicondylitis Duration: NA	Group I (*n* = 30): ESWT Age (years): 47.6 ± 7.66 Group II (*n* = 30): UST Age (years): 43.9 ± 9.44	ESWT: 2000 shocks—5 Hz, 2.5 bars, three sessions for two weeks Site: the most tender point on the lateral epicondyle	1 MHz, 1.5 W/cm^2^, continuous mode, 5 min, ten sessions held on weekdays over 2 weeks Site: lateral elbow	pain (VAS), CET SMI, PRTEE scores (pain, function)	post-treatment	Both groups showed significant improvements in PRTEE and VAS scores after treatment. ESWT had greater post-treatment score reductions than UST

ESWT—extracorporeal shock wave therapy; RSWT—radial shock wave therapy; UST—ultrasound therapy; VAS—Visual Analogue Scale; PRTEE—Patient-Rated Tennis Elbow Evaluation Scale; LE—Lateral epicondylitis; NA—not applicable; SF-36—Study 36-Item Short Form Health Survey; DASH—Disabilities of the Arm, Shoulder and Hand; CET—common extensor tendon; SMI—superb microvascular imaging.

**Table 2:** Characteristics of included studies in which ESWT and ultrasound therapy were used in combination with other conservative therapies for tendinopathy.

Reference	Diagnosis	Groups	ESWT Intervention	UST Intervention	Outcome Measure	Follow-Up	Results
Özmen et al., 2021 [31] (Turkey)	Lateral epicondylitis Duration (months): >3 Group I: 8.07 ± 8.76 Group II: 2.92 ± 2.98	Group I (*n* = 14): ESWT + TENS + hot pack Age (years): 48.36 ± 11.51 Group II (*n* = 13): UST + TENS + hot pack Age (years): 49.62 ± 10.20	ESWT: 1500 shocks—4 Hz, 1.4 bars, 0.22 mJ/mm^2^, 3 sessions for 2 weeks Site: NA	1 MHz, 1 W/cm^2^ for 3 min, ten sessions held on weekdays over 2 weeks for 2 weeks. Site: NA	PRTEE scores (pain, function), pain (VAS at rest, VAS at ADL), grip strength (Jamar Dynamometer), CET thickness (sonographic imaging)	2 and 8 weeks after the final treatment session	The UST, and ESWT are effective in reducing pain and improving functionality. However, none of these treatment methods were found to be superior to others in reducing the pain and improving functionality
Król et al., 2024 [33] (Poland)	Lateral epicondylitis Duration (months): ≥3 Group I: 7.1 ± 5.25 Group II: 8.1 ± 8.25	Group I (*n* = 20): FSWT + deep friction massage. Age (years): 47.15 ± 8.56 Group II (*n* = 20): UST + deep friction massage. Age (years): 46.9 ± 8.5	FSWT: 2000 shocks—4 Hz, 0.2 mJ/mm^2^, 3 sessions, once a week. Site: the most painful point of the lateral epicondyle	3 MHz, 0.5 W/cm^2^, pulsed mode (20%), 5 min, ten sessions held on weekdays over 2 weeks Site: the most painful point of the lateral epicondyle	pain intensity during activity (NRS), PRTEE scores (pain, function), wrist muscle strength (dynamometer)	1, 3, 6 and 12 weeks after the final treatment session	Both FSWT and UST combined with deep friction massage were effective for lateral epicondylitis. FSWT led to significantly greater pain relief and functional improvement than UST, while gains in wrist extensor and grip strength were similar for both treatments.
Ranjithkumar et al., 2025 [32] (India)	Rotator cuff tendinopathy Duration (weeks): >4	Group I (*n* = 25): ESWT + dynamic loading exercises Age (years): 30–50 Group II (*n* = 25): US + dynamic loading exercises Age (years): 30–60	ESWT: 0.2 mJ/mm^2^, 2000 shocks, 4 sessions; once a week. Site: supraspinatus tendon, subscapularis tendon, infraspinatus and teres minor tendons	1 MHz, pulsed mode 20% to 50%, 8 min, 20 sessions held on weekdays over 4 weeks Site: over the shoulder joint	pain (NRS), function (CMS score), activities of daily living (ADL), ROM, power (CMS Subscales)	1, 2, 3 and 4 weeks after the final treatment session	Low-energy ESWT combined with dynamic rotator cuff exercises is the most effective treatment for rotator cuff tendinopathy, offering superior improvements in muscle thickness, shoulder function, and pain relief compared to UST

ESWT—extracorporeal shock wave therapy; FSWT—focused shock wave therapy; UST—ultrasound therapy; TENS—Transcutaneous Electrical Nerve Stimulation; ADL—activities of daily living; PRTEE—Patient-Rated Tennis Elbow Evaluation Scale; CET—common extensor tendon; NRS—Numerical rating scale; NA—not applicable; CMS—The Constant–Murley Score; ROM—Range of Motion; VAS—Visual Analogue Scale.

**Table 3:** Characteristics of included studies in which ESWT and ultrasound therapy were used in combination with painkillers and anti-inflammatory drugs.

Reference	Diagnosis	Groups	ESWT Intervention	UST Intervention	Outcome Measure	Follow-Up	Results
Kubot et al., 2017 [34] (Poland)	Lateral epicondylitis Duration: NA	Group I (*n* = 30): RSWT Age (years): 47.6 ± 7.66 Group II (*n* = 30): UST Age (years): 43.9 ± 9.44 Patients were allowed to take pain medication during the study	RSWT first phase: 2000 shocks—8 Hz, 1.5–2.5 bars, 3 sessions, once a week Site: the region of the lateral epicondyle RSWT second phase: 2000 shocks—8 Hz, 2.5–3.5 bars, 3 sessions, once a week Site: the extensor carpi radialis brevis trigger points	First phase: 1 MHz, 0.5 W/cm^2^, pulsed mode (50%), 3 min, ten sessions held on weekdays over 2 weeks Site: around the lateral humeral epicondyle Second phase: 1 MHz, 0.5 W/cm^2^, pulsed mode (50%), 2 min, ten sessions held on weekdays over 2 weeks Site: the extensor carpi radialis brevis trigger points	pain (VAS, Leitinen questionnaire), need for pain medication	post-treatment and 8 weeks after the final treatment session	Both RSWT and UST cause a reduction in the intensity and frequency of pain reducing the need for pain medication and improving the function of the treated upper limb. UST is less effective than RSWT
Carlisi et al., 2019 [35] (Italy)	Gluteal tendinopathy Duration (weeks): >6	Group I (*n* = 26): ESWT Age (years): 61 ± 9.18 Group II (*n* = 24): UST Age (years): 61.5 ± 9.52 When needed, the patients were allowed to take paracetamol in a daily dose of up to 1000 mg or ibuprofen 400 mg	FSWT: 1800 shocks—4 Hz, 0.15 mJ/mm^2^, 3 sessions, once a week Site: the enthesis of the gluteus medius on the anterior part of the greater trochanter’s lateral facet	1 MHz, 1.5 W/cm^2^, continuous mode, 10 min, ten sessions held on weekdays over 2 weeks Site: the enthesis of the gluteus medius on the anterior part of the greater trochanter’s lateral facet	pain (NRS), LEFS scale	2 and 6 months after the final treatment session	FSWT reduces pain in the short and mid-term, with functional gains similar to UST
Dedes et al., 2020 [36] (Greece)	Lateral epicondylitis Duration: NA	Group I (*n* = 117): ESWT Age: NA Group II (*n* = 63): UST Age: NA 81 patients used local application of NSAIDs in the form of gels and creams or used oral NSAIDs	ESWT (for the initial session): 2000 shocks—21 Hz, 1.8 bars ESWT (for all the remaining sessions): 1500 shocks—15 Hz, 1.6 bars, 97 patients received three treatments, 11 received four treatments, and nine received five treatments, once a week Site: along the extensor tendons	3 MHz, 2 W/cm^2^, 10 sessions, three times a week Site: along the extensor tendons	pain (UoPPFQ), functional impairment, and quality of life impairment (five-point Likert scale)	post-treatment and 4 weeks after the final treatment session	Both ESWT and UST were effective for lateral epicondylitis, but shockwave therapy was more effective
Stania et al., 2023a [26] (Poland)	Achilles tendinopathy Duration (months): >3 Group I: 8.84 ± 8.68 Group II: 9.07 ± 7.64	Group I (*n* = 13): RSWT Age (years): 42 ± 11.42 Group II (*n* = 13): UST Age (years): 36.69 ± 11.57 When needed, the patients were allowed to take paracetamol in a daily dose of up to 4000 mg.	RSWT: 2000 shocks—10 Hz, 3 bars on Achilles tendon then 2000 shocks, on gastrocnemius muscle, 3 sessions, once a week	3 MHz, 1 W/cm^2^, pulsed mode (50%), each square centimeter was exposed to ultrasonic energy for 2 min, ten sessions held on weekdays over 2 weeks Site: Achilles tendon	pain (VAS), static posturographic measurement (force platforms)	1 and 6 weeks after the final treatment session	RSWT outperformed UST in easing activity-related pain and was linked to better postural control in Achilles tendinopathy. Center-of-pressure trajectories in the sagittal plane were greater in the non-affected limb
Stania et al., 2023b [27] (Poland)	Achilles tendinopathy Duration (months): >3 Group I: 8.84 ± 8.68 Group II: 9.07 ± 7.64	Group I (*n* = 13): RSWT Age (years): 42 ± 11.42 Group II (*n* = 13): UST Age (years): 36.69 ± 11.57 When needed, the patients were allowed to take paracetamol in a daily dose of up to 4000 mg.	RSWT: 2000 shocks—10 Hz, 3 bars on Achilles tendon then 2000 shocks, on gastrocnemius muscle, 3 sessions, once a week	3 MHz, 1 W/cm^2^, pulsed mode (50%), each square centimeter was exposed to ultrasonic energy for 2 min, ten sessions held on weekdays over 2 weeks Site: Achilles tendon	dynamic posturography during step-up and step-down tasks (force platforms)	1 and 6 weeks after the final treatment session	Posturographic testing during step-up/step-down showed no therapeutic superiority among the two treatments for non-insertional Achilles tendinopathy
Stania et al., 2024 [14] (Poland)	Achilles tendinopathy Duration (months): >3 Group I: 8.84 ± 8.68 Group II: 9.07 ± 7.64	Group I (*n* = 13): RSWT Age (years): 42 ± 11.42 Group II (*n* = 13): UST Age (years): 36.69 ± 11.57 When needed, the patients were allowed to take paracetamol in a daily dose of up to 4000 mg.	RSWT: 2000 shocks—10 Hz, 3 bars on Achilles tendon then 2000 shocks, on gastrocnemius muscle, 3 sessions, once a week	3 MHz, 1 W/cm^2^, pulsed mode (50%), each square centimeter was exposed to ultrasonic energy for 2 min, ten sessions held on weekdays over 2 weeks Site: Achilles tendon	VISA-A questionnaire, posturographic measurements of step initiation under two different conditions (non-perturbed and perturbed transit) (force platforms)	1 and 6 weeks after the final treatment session	RSWT outperformed UST in reducing pain and improving function in non-insertional Achilles tendinopathy, supporting its clinical use
Król et al., 2024 [37] (Poland)	Achilles tendinopathy Duration (months): >3 Group I: 8.84 ± 8.68 Group II: 9.07 ± 7.64	Group I (*n* = 13): RSWT Age (years): 42 ± 11.42 Group II (*n* = 13): UST Age (years): 36.69 ± 11.57 When needed, the patients were allowed to take paracetamol in a daily dose of up to 4000 mg.	RSWT: 2000 shocks—10 Hz, 3 bars on Achilles tendon then 2000 shocks, on gastrocnemius muscle, 3 sessions, once a week	3 MHz, 1 W/cm^2^, pulsed mode (50%), each square centimeter was exposed to ultrasonic energy for 2 min, ten sessions held on weekdays over 2 weeks Site: Achilles tendon	posturographic measurement with rambling-trembling decomposition and sample entropy (force platforms)	1 and 6 weeks after the final treatment session	Trembling trajectories were smaller in affected limbs. The UST group showed larger sway paths than the RSWT group. Postural control was worse with eyes closed, while sample entropy was unaffected by therapy, time, or limb condition

ESWT—extracorporeal shock wave therapy; FSWT—focused shock wave therapy; RSWT—radial shock wave therapy; UST—ultrasound therapy; NA—not applicable; VAS—Visual Analogue Scale; NRS—Numerical Rating Scale; LEFS—lower extremity functional scale; VISA-A—Victorian Institute of Sport Assessment–Achilles; UoPPFQ—University of Peloponnese Pain, Functionality and Quality of Life Questionnaire; NSAIDs—Nonsteroidal anti-inflammatory drug.

## Data Availability

All data supporting the findings of this systematic review are available within the manuscript.

## Supplementary Materials

The following supporting information can be downloaded at: https://www.mdpi.com/article/10.3390/jcm15052007/s1, Figure S1: Forest plot comparing pain intensity between ESWT and ultrasound therapy, both combined with analgesics and anti-inflammatory drugs by ESWT type (RSWT vs. FSWT); Figure S2: Forest plot comparing pain intensity between ESWT and ultrasound therapy, both combined with analgesics and anti-inflammatory drugs by ultrasound mode (continuous vs. pulsed); Table S1: Detailed search strategy; Table S2: Randomized controlled trials on the efficacy of EWST and ultrasound therapy for tendinopathy, rated using the Physiotherapy Evidence Database (PEDro) scale; Table S3: Summary of findings using the Grading of Recommendations Assessment, Development, and Evaluation (GRADE) system; Table S4: PRISMA 2020 Checklist.
