# Supplementary material for: Shockwave or Ultrasound Therapy for Tendinopathy? A Systematic Review and Meta-Analysis
Source: J Clin Med. 2026 Mar 5;15(5):2007. doi: 10.3390/jcm15052007 (PMC12985698; doi:10.3390/jcm15052007)
Supplement: Supplementary file 1 [file jcm-15-02007-s001.zip › Supplementary figure 1..pdf]

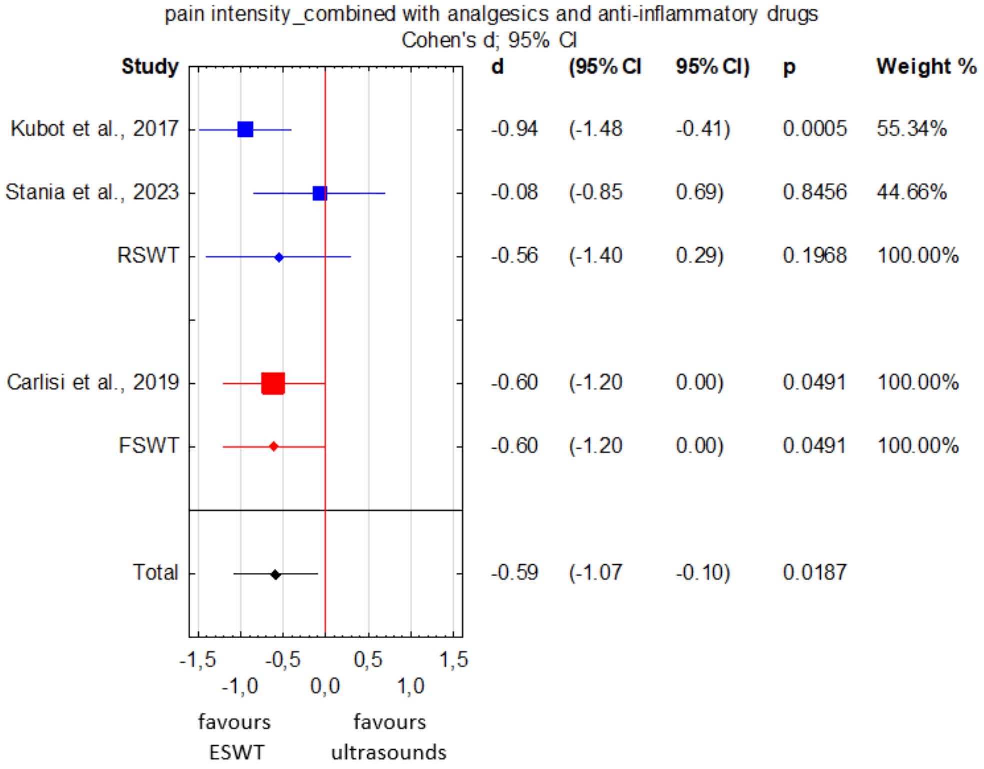

Supplementary Fig. 1. Forest plot comparing pain intensity between ESWT and ultrasound therapy, both combined with analgesics and anti-inflammatory drugs by ESWT type (RSWT vs. FSWT).
