# Supplementary material for: Shockwave or Ultrasound Therapy for Tendinopathy? A Systematic Review and Meta-Analysis
Source: J Clin Med. 2026 Mar 5;15(5):2007. doi: 10.3390/jcm15052007 (PMC12985698; doi:10.3390/jcm15052007)
Supplement: Supplementary file 1 [file jcm-15-02007-s001.zip › Supplementary table 1..pdf]

Supplementary material

Supplementary Table 1. Detailed search strategy

**PubMed**<br>(((shock wave[tiab] OR shock-wave[tiab] OR shockwave[tiab] OR shockwaves[tiab] OR extracorporeal[tiab])) AND (ultrasound[tiab] OR ultrasonic[tiab] OR ultrasonics[tiab] OR sonotherapy[tiab]) AND (tendinopathy[tiab] OR enthesopathy[tiab] OR tendonitis[tiab] OR tendonopathy[tiab] OR tendinosis[tiab] OR epicondylitis[tiab] OR tennis elbow[tiab] OR golfer's elbow[tiab] OR lateral epicondylitis[tiab] OR medial epicondylitis[tiab] OR humeral epicondylitis[tiab]))
**Embase**<br>('tendinopathy'/exp OR 'tendinopathy' OR 'tendinitis'/exp OR 'tendinitis' OR 'enthesopathy'/exp OR 'enthesopathy' OR 'tennis elbow'/exp OR 'tennis elbow' OR 'golfers elbow' OR 'epicondylitis'/exp OR 'epicondylitis') AND ('shock wave therapy'/exp OR 'shock wave therapy' OR 'shockwave'/exp OR 'shockwave') AND ('ultrasound therapy'/exp OR 'ultrasound therapy' OR 'ultrasonic'/exp OR 'ultrasonic')
**EBSCOhost**<br>TX (shockwave therapy or shock wave therapy or shock-wave therapy or extracorporeal shockwave) AND TX (ultrasound therapy or ultrasound treatment or therapeutic ultrasound) AND TX (tendinopathy or tendonitis or tendonopathy or tendinosis or tendinosis)
**Ovid MEDLINE**<br>Ovid MEDLINE(R) ALL <1946 to March 25, 2025><br>1 shock wave therapy.mp. or exp Extracorporeal Shockwave Therapy/ 2708<br>2 ultrasound therapy.mp. or exp Ultrasonic Therapy/ 15890<br>3 1 and 2 1395<br>4 exp Tendinopathy/ or exp Elbow Tendinopathy/ or tendinopathy.mp. 18541<br>5 3 and 4 209
