# Supplementary material for: Shockwave or Ultrasound Therapy for Tendinopathy? A Systematic Review and Meta-Analysis
Source: J Clin Med. 2026 Mar 5;15(5):2007. doi: 10.3390/jcm15052007 (PMC12985698; doi:10.3390/jcm15052007)
Supplement: Supplementary file 1 [file jcm-15-02007-s001.zip › Supplementary table 2..pdf]

Supplementary Table 2. Randomized controlled trials on the efficacy of EWST and ultrasound therapy for tendinopathy, rated using the Physiotherapy Evidence Database (PEDro) scale.

Reference	Eligibility criteria specified	Subjects randomly allocated to groups	Allocation concealed	Groups similar at baseline	Blinding of all subjects	Blinding of all therapists	Blinding assessors	> 85% follow up	Intention-to-treat analysis	Between-group statistical comparison	Point and variability measures	Score*
Lizis et al., 2015	+	+	+	+	-	-	-	+	+	+	+	**7**
Kubot et al., 2017	+	+	-	-	-	-	-	+	+	+	+	**5**
Białek et al., 2018	+	+	-	+	-	-	-	+	+	+	+	**6**
Yalvaç et al., 2018	+	+	-	+	-	-	+	-	-	+	+	**5**
Carlisi et al., 2019	+	+	+	+	-	-	+	+	+	+	+	**8**
Dedes et al., 2020	+	+	-	-	-	-	-	+	+	+	+	**5**
Özmen et al., 2021	+	+	-	+	-	-	-	+	+	+	+	**6**
Ustabaşıoğlu et al., 2023	+	+	-	+	-	-	+	+	-	+	+	**6**
Stania et al., 2023a	+	+	+	+	-	-	+	+	-	+	+	**7**
Stania et al., 2023b	+	+	+	+	-	-	+	+	-	+	+	**7**
Stania et al., 2024	+	+	+	+	-	-	+	+	-	+	+	**7**
Król et al., 2024a	+	+	+	+	-	-	+	+	-	+	+	**7**
Król et al., 2024b	+	+	+	+	-	-	+	+	-	+	+	**7**
Ranjithkumar et al., 2025	+	+	+	+	-	-	-	+	+	+	+	**7**

*The *Eligibility criteria* item is not included in the PEDro score calculation.
