# Supplementary material for: Shockwave or Ultrasound Therapy for Tendinopathy? A Systematic Review and Meta-Analysis
Source: J Clin Med. 2026 Mar 5;15(5):2007. doi: 10.3390/jcm15052007 (PMC12985698; doi:10.3390/jcm15052007)
Supplement: Supplementary file 1 [file jcm-15-02007-s001.zip › Supplementary table 3..pdf]

Supplementary Table 3. Summary of findings using the Grading of Recommendations Assessment, Development, and Evaluation (GRADE) system. Question: Should extracorporeal shock wave therapy (ESWT) vs. Ultrasound be used for tendinopathy?

Certainty assessment							№ of patients		Effect		Certainty	Importance
№ of studies	Study design	Risk of bias	Inconsistency	Indirectness	Imprecision	Other considerations	ESWT	ultrasound therapy	Relative (95% CI)	Absolute (95% CI)		
**Rest pain_monotherapy**												
2	randomised trials	very serious[a]	serious[b]	not serious	serious[c]	none	55	55	-	MD **1.51 lower** (2.71 lower to 0.31 lower)	⊕◯◯◯ Very low[a,b,c]	NOT IMPORTANT
**Pain_therapy in combination with painkillers and anti-inflammatory drugs**												
3	randomised trials	very serious[a]	not serious	not serious	serious[c]	none	67	64	-	SMD **0.6 SD lower** (1.07 lower to 0.14 lower)	⊕◯◯◯ Very low[a,c]	NOT IMPORTANT
**PRTEE score_monotherapy**												
2	randomised trials	very serious[d]	serious[e]	not serious	serious[f]	none	50	54	-	MD **1.06 lower** (11.06 lower to 8.94 higher)	⊕◯◯◯ Very low[d,e,f]	NOT IMPORTANT
**PRTEE score_combination therapy**												
2	randomised trials	very serious[a]	not serious	not serious	serious[f]	none	34	33	-	MD **0.46 higher** (10.22 lower to 11.15 higher)	⊕◯◯◯ Very low[a,f]	NOT IMPORTANT

CI: confidence interval; **MD:** mean difference; **SMD:** standardised mean difference. Explanations: a) High risk of bias due to lack of blinding of patients, therapists, and assessors; some concerns regarding allocation concealment; and no intention-to-treat analysis; b) significant heterogeneity was observed (I2=89.80%; p=0.0017); c) small sample size; d) High risk of bias due to lack of blinding of patients, therapists, and assessors, as well as incomplete outcome data; some concerns regarding allocation concealment; and absence of an intention-to-treat analysis; e) significant heterogeneity was observed (I2=75,82%; p=0.04); f) small sample size, wide confidence intervals around estimates of effect
